# Trigger Warning: How Modern Diet, Lifestyle, and Environment Pull the Trigger on Autosomal Dominant Polycystic Kidney Disease Progression

**DOI:** 10.3390/nu16193281

**Published:** 2024-09-27

**Authors:** Melina Messing, Jacob A. Torres, Nickolas Holznecht, Thomas Weimbs

**Affiliations:** Department of Molecular, Cellular, and Developmental Biology, University of California Santa Barbara, Santa Barbara, CA 93106, USA; mmessing@ucsb.edu (M.M.); jacobtorres@ucsb.edu (J.A.T.); nholznecht@ucsb.edu (N.H.)

**Keywords:** polycystic kidney disease, chronic kidney disease, disease progression, nutrition, dietary guidelines

## Abstract

Understanding chronic kidney disease (CKD) through the lens of evolutionary biology highlights the mismatch between our Paleolithic-optimized genes and modern diets, which led to the dramatically increased prevalence of CKD in modern societies. In particular, the Standard American Diet (SAD), high in carbohydrates and ultra-processed foods, causes conditions like type 2 diabetes (T2D), chronic inflammation, and hypertension, leading to CKD. Autosomal dominant polycystic kidney disease (ADPKD), a genetic form of CKD, is characterized by progressive renal cystogenesis that leads to renal failure. This review challenges the fatalistic view of ADPKD as solely a genetic disease. We argue that, just like non-genetic CKD, modern dietary practices, lifestyle, and environmental exposures initiate and accelerate ADPKD progression. Evidence shows that carbohydrate overconsumption, hyperglycemia, and insulin resistance significantly impact renal health. Additionally, factors like dehydration, electrolyte imbalances, nephrotoxin exposure, gastrointestinal dysbiosis, and renal microcrystal formation exacerbate ADPKD. Conversely, carbohydrate restriction, ketogenic metabolic therapy (KMT), and antagonizing the lithogenic risk show promise in slowing ADPKD progression. Addressing disease triggers through dietary modifications and lifestyle changes offers a conservative, non-pharmacological strategy for disease modification in ADPKD. This comprehensive review underscores the urgency of integrating diet and lifestyle factors into the clinical management of ADPKD to mitigate disease progression, improve patient outcomes, and offer therapeutic choices that can be implemented worldwide at low or no cost to healthcare payers and patients.

## 1. Introduction

Nothing in biology makes sense except in the light of evolution [1]. This is not different when it comes to understanding chronic kidney disease (CKD). Evolutionary biology dictates that, just as for other non-domesticated animals today, CKD would have been a rare occurrence in our ancestors during human evolution. Kidneys are responsible for eliminating metabolites and toxins that are largely controlled by diet and environment. Therefore, kidneys are impacted by changes in nutrition and environmental exposures. The genes controlling the renal physiology of today’s Homo sapiens have evolved over a few million years during the Paleolithic to be optimized based on our ancestral nutrition during this time [2,3,4,5]. Our genetic makeup has remained practically unchanged since the start of the agricultural revolution about 10 K years ago, which caused a substantial shift in human nutrition with the introduction of novel staple foods such as grains and other plant foods with high-glycemic loads. An even more radical shift in human nutrition has occurred since the increasing availability of novel foods and ultra-processed foods during the last few decades. These rapid changes in nutrition were not accompanied by corresponding genetic changes that would have allowed for physiological adaptation [2,3,4,5].

Ancestral diets of our hunter-gatherer predecessors, for which our kidneys are still optimized, differed substantially from today’s Standard American Diet (SAD) and even from the diets advocated by today’s governments, such as the Dietary Guidelines for Americans [6]. It should, therefore, be no surprise that, in modern times, CKD has become increasingly prevalent globally and has reached pandemic proportions [7]. In 2023, 14% of US adults were estimated to have CKD [8]. The bulk of the increase in the prevalence of CKD is due to a similarly dramatic increase in poor metabolic health during the past decades, leading to epidemics of type 2 diabetes (T2D) and hypertension, the known leading causes of CKD. These morbidities are primarily caused by inappropriate nutrition, especially overconsumption of carbohydrates (sugars and starches), leading to persistent hyperglycemia, hyperinsulinemia, and insulin resistance [9]. Mechanistically, many associated processes lead to persistent renal damage, such as direct vascular damage due to toxic effects of glucose, hypertensive damage, renal inflammation, and numerous other mechanisms, all eventually leading to progressive CKD.

Since most cases of CKD are caused by toxic effects (primarily of glucose and indirect effects caused by hyperglycemia and hyperinsulinemia), CKD is technically not a “disease” but a “toxicity”. Therefore, appropriate medical intervention should start with the elimination of exposure to the toxic agent. However, this is not current clinical practice and, unfortunately, most patients are advised to remain on the high-carbohydrate diets that caused their CKD in the first place. The chronic kidney “toxicity” is usually merely managed by medicating for symptoms while leaving the toxic exposure unmitigated.

In addition to purely “diet-induced” forms of CKD, which are by far the most common forms today, there are also genetically determined forms of CKD. The most common genetic form is autosomal dominant polycystic kidney disease (ADPKD). The progression of CKD in ADPKD involves largely the same mechanisms as in CKD induced by inappropriate diet alone, including renal inflammation, fibrosis, and vascular and tubular injury. It includes largely the same molecular players, such as numerous signaling pathways, growth factors, and cytokines. A notable distinction is that ADPKD is additionally characterized by the growth of large, fluid-filled cysts, which result in gross organ enlargement that is not typically seen in other forms of CKD.

As a genetic disease, ADPKD has historically been regarded as a relentlessly progressive disease that is not caused or influenced by external factors such as diet and lifestyle. Due to this fatalistic concept, clinical interventions for ADPKD are currently aimed at pharmacological management rather than prevention. Besides dialysis and kidney transplantation after reaching End Stage Renal Disease (ESRD), only one drug is approved for the treatment of ADPKD. Tolvaptan modestly slows CKD progression in ADPKD but has significant side effects and toxicities and is only available to a subset of patients worldwide due to high cost and strict eligibility criteria [10,11,12].

Despite being transmitted in a straightforward Mendelian manner, ADPKD families show remarkable variability in disease expression and progression among affected individuals [13]. In clinical practice, ADPKD presents a wide spectrum of severity and age at onset, even within the same family. This intrafamilial variability contributes to significant challenges in predicting individual disease trajectories and strongly suggests that, despite the simple monogenic basis, external factors, such as environmental and nutritional factors, may exacerbate or ameliorate pathophysiological processes in affected individuals. Emerging research has confirmed such disease-modifying effects by external factors. However, current clinical practice is lagging behind and does not yet target modifiable external factors that could alter disease progression.

In this review, we will discuss cumulative and recent advances in ADPKD research that have led to the conclusion that the current concept of regarding ADPKD simply as a genetic disease with inevitable and unstoppable progression is likely incorrect. We will argue that diet and lifestyle factors associated with modern diets are disease-modifying (worsening) in ADPKD involving the same mechanisms that lead to most non-genetic forms of CKD. Conversely, mitigating changes in diet and lifestyle factors can be disease-modifying (improving) and prevent, or at least significantly slow, the progression of ADPKD. The main emerging premise is that the initiation and progression of CKD in ADPKD are triggered by diet and lifestyle factors, just as they are in purely diet-induced forms of CKD. While the kidneys in individuals with ADPKD may be more sensitive to these triggers, the same triggers are nevertheless required to induce CKD.

## 2. Genetic Foundations of ADPKD

Mutations in either of two genes underlie most cases of ADPKD: the PKD1 or PKD2 genes [14]. They encode the proteins polycystin-1 (PC1), a large membrane receptor involved in cell–cell and cell–matrix interactions, and polycystin-2 (PC2), a calcium-permeable ion channel. PC1 and PC2 interact to form a functional complex that is essential for normal renal tubular development and function. Mutations in these genes disrupt this complex, leading to abnormal cellular signaling, cell proliferation, and fluid secretion, all of which contribute to the formation and growth of renal cysts. Key molecular pathways implicated in ADPKD pathogenesis include the mammalian target of rapamycin (mTOR), cyclic AMP (cAMP), and Wnt signaling pathways/but numerous others have been identified [15]. Inflammatory processes are also prominent, with increased levels of pro-inflammatory cytokines and massive immune cell infiltration, contributing to renal fibrosis and disease progression.

The majority of ADPKD cases are due to mutations in PKD1, which tend to result in a more severe disease phenotype with earlier onset and faster progression compared to mutations in PKD2. The mutations in these genes are highly heterogeneous, including missense, nonsense, frameshift, and splice-site mutations, which can lead to either complete loss of function or a dominant-negative effect, exacerbating the progression of the disease [14]. In addition to PKD1 or PKD2, modifier genes, which do not cause PKD on their own, may modulate the phenotypic expression of the disease and are thought to contribute to variability in clinical outcomes even among individuals with the same primary mutation [16]. This suggests that the efficacy of lifestyle and dietary approaches may vary depending on the genetic mutation. For instance, individuals with truncating PKD1 mutations may require more rigorous or earlier intervention to achieve similar benefits compared to those with minor PKD2 mutations, and modifier genes may further influence the responsiveness to these interventions. However, while genetics undoubtedly play a role in disease variability, we argue in this review that lifestyle and environmental factors are major modifiable drivers of ADPKD progression. Dietary interventions are likely to be effective across genetic backgrounds and modifier genes, making them a powerful strategy to slow or even reverse disease progression regardless of the specific genetic mutation involved.

## 3. Genes versus Triggers

**Figure 1:**
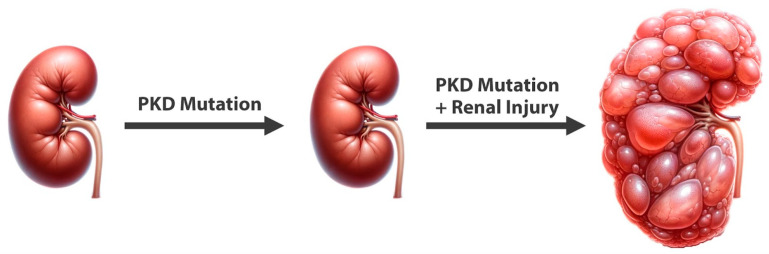
**Model of renal injury-mediated ADPKD development.** In this model, PKD mutations are not enough to initiate disease. Renal injury (that occurs in response to many dietary, lifestyle, and environmental triggers) is required to drive ADPKD development.

The saying that “*genes load the gun, but lifestyle pulls the trigger*” is conventional wisdom in medicine and widely accepted for many of today’s non-communicable diseases, including cancer, cardiovascular disease, diabetes, diabetic nephropathy, and many more. However, among healthcare practitioners, ADPKD is not considered to fall under this wisdom even though animal experiments have already clearly demonstrated this years ago. A series of landmark findings showed that inactivation of the *Pkd1* gene in mature mice is largely inconsequential and does not lead to polycystic kidney disease for a very long time [17]. However, if the animals are challenged with any form of renal injury subsequent to the *Pkd1* gene deletion, this injury triggers rapid renal cystic disease [18]. These findings led to the concept that renal injury is required for cystogenesis and CKD progression in ADPKD, in addition to the underlying gene mutation [18]. The idea is that renal injury triggers innate renal repair mechanisms that would normally resolve acute kidney injuries (AKI). However, in ADPKD, the renal repair mechanisms—once triggered—will go out of hand and continue persistently (Figure 1).

In rodent experiments, it has been shown that various forms of renal injury lead to rapid onset of polycystic kidney disease in genetically predisposed animals, including injury by hyperglycemia [19], nephrotoxins [20], ischemia-reperfusion injury [21], and injury by renal tubular microcrystal deposition such as calcium oxalate and calcium phosphate [22] (Figure 1). Given the wide variation of these forms of injury, it is reasonable to conclude that almost any form of renal injury may trigger cystogenesis and progression of polycystic kidney disease in animals, including humans, that are genetically predisposed.

## 4. Implications for Dietary Guidelines and Interventions

As discussed below, there is good evidence that various forms of renal injury—especially those caused by dietary factors—act as triggers and worsen disease progression in ADPKD. These factors are already well known to cause AKI and CKD in humans, even without any underlying genetic mutations. It is more than plausible that individuals with ADPKD, who are genetically predisposed to “overreact” to renal injury, are highly sensitive to these triggers.

Unfortunately, since the concept of “genes load the gun, but lifestyle pulls the trigger” is not yet commonly applied to ADPKD in clinical practice, there is an absence of clear dietary recommendations that would address potential triggers. The absence of such dietary recommendations (“there is nothing you can do”) leads most individuals with ADPKD in the US to default to the Standard American Diet (SAD). The SAD is characterized by very high carbohydrate consumption of sugars and starches (45–65% of calories per the official Dietary Guidelines for Americans, DGA), which frequently leads to persistent hyperglycemia, hyperinsulinemia, insulin resistance, obesity, hypertension, chronic inflammation, diabetes, and damage to numerous tissues and organs, including the kidneys, leading to CKD. The SAD is also characterized by very high consumption of ultra-processed foods (UPFs). A recent meta-analysis found that consumption of UPFs represents up to 80% of total caloric intake in the US [23]. Consumption of UPFs usually starts from the earliest age—with infant formulas and ready-made baby foods—and continues throughout all stages of life. The SAD also often leads to hyperuricemia (high serum uric acid (UA) levels) primarily due to high fructose/sugar consumption, greatly increasing the risk of gout, hypertension, CKD, and kidney stones. The SAD also has a significant impact on the gastrointestinal microbiome, causing significant alterations in microbial metabolism, dysbiosis, intestinal permeability, and systemic inflammation [24,25].

Currently, even some PKD patient advocacy groups recommend to their constituents that “*Currently no specific diet has been proven to make your polycystic kidneys better or keep them from getting worse*” (https://pkdcure.org/living-with-pkd/nutrition (accessed on 26 August 2024)). While this statement may not be technically incorrect, it ignores the fact that no clinical trial has ever shown that the SAD is beneficial in ADPKD. Since the effect of such a statement is that individuals with ADPKD will—more likely than not—default to the SAD, it is essentially an endorsement of the SAD, stands in the way of better clinical practice, and may endanger individuals with ADPKD.

**Figure 2:**
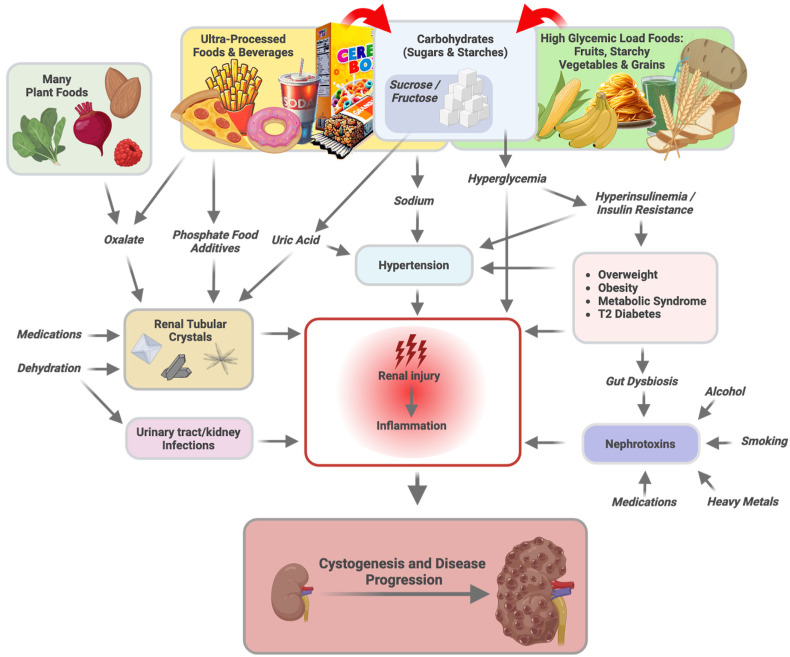
Summary of dietary, lifestyle, and environmental triggers that accelerate ADPKD progression.

This review is not medical advice but rather a detailed discussion of the factors and mechanisms controlled by diet and environment that can impact ADPKD progression (Figure 2) and underscores the urgent need to bring these concepts to the forefront of ADPKD clinical management.

## 5. Carbohydrates

### 5.1. Carbohydrate Overconsumption, Persistent Hyperglycemia, and Renal Health

Carbohydrates are probably the clearest case of a trigger that worsens ADPKD progression. Persistent hyperglycemia due to carbohydrate overconsumption is the most common and well-recognized driver of non-genetic CKD in the form of diabetic nephropathy. Renal injury occurs via direct toxic effects of glucose and numerous indirect effects in response to persistent hyperglycemia, including hyperinsulinemia and insulin resistance, hypertension, chronic inflammation, and hyperuricemia (Figure 2) [26]. In combination, persistent hyperglycemia affects not only glomeruli but also tubule epithelial and interstitial cells [26]. The presence of T2D in individuals with ADPKD is associated with larger renal volumes, worsened hypertension, and earlier death than in those individuals with ADPKD alone [27].

Similarly, overweight, obesity, and visceral adiposity—common markers of poor metabolic health due to persistent hyperglycemia and insulin resistance—are associated with worse ADPKD progression [28,29,30]. From 2009–2016, the prevalence of metabolic health among Americans has decreased from an already low level of 19.9% to a vanishingly low level of 12.2% [31], meaning that 87.8% of Americans are metabolically unhealthy. Given this trend, any possible effect of poor metabolic health on ADPKD progression should be alarming to patients and their healthcare practitioners.

Mechanistic animal experiments have demonstrated that hyperglycemia not only leads to renal structural and functional damage but also triggers cystogenesis and PKD disease progression in a genetically predisposed mouse model [19]. Altogether, it is clear that diet and lifestyle factors that lead to poor metabolic health and, eventually, T2D are triggers of ADPKD progression. The underlying mechanisms are likely largely identical to those that lead to diabetic nephropathy. Dietary carbohydrates that contribute to a glycemic load encompass (1) simple sugars in mono- or disaccharide form (e.g., glucose, fructose, sucrose) and (2) starches, which are polymers of glucose. It is a common misconception that starches—often referred to as “complex carbohydrates”—have a substantially lower glycemic load than simple sugars. Consuming healthy-sounding foods such as whole-grain bread or brown rice contributes to hyperglycemia just as much as consuming a banana or candy [32]. One distinction, however, is that starch, in contrast to ordinary sugar (sucrose), does not contain fructose. Fructose-containing sugars are contained naturally in fruits and are also added, in refined form, to most UPFs. The metabolism of fructose leads to additional direct and indirect challenges for kidneys, including hypertension (discussed in this section) as well as hyperuricemia and gastrointestinal dysbiosis (further discussed below) [33,34].

### 5.2. Hyperinsulinemia, Fructose, and Hypertension

Chronic hypertension mediates renal injury primarily through direct damage of renal capillaries and, over time, leads to inflammation and fibrosis. The deleterious effect of hypertension on ADPKD progression is well established [35], and hypertension is routinely managed with anti-hypertensive medications and/or one of the only commonly accepted dietary recommendations: a reduction in sodium intake. Yet, the clear connection between hypertension and carbohydrates, specifically hyperinsulinemia and high-fructose consumption, is widely ignored [36,37]. Hyperinsulinemia, characterized by elevated blood insulin, which is usually a direct result of a diet chronically high in carbohydrates, has well-established mechanistic links to hypertension [38]. Insulin directly stimulates sodium reabsorption in renal tubules by enhancing the activity of sodium transporters while also activating the sympathetic nervous system and the renin-angiotensin-aldosterone system (RAAS), both of which increase sodium retention and blood pressure [38,39]. Additionally, hyperinsulinemia impairs endothelial function, reducing nitric oxide production and increasing endothelin-1 levels, leading to vasoconstriction and further sodium retention [40,41].

Fructose consumption, too, leads to several overlapping effects that culminate in increased blood pressure, including increased intestinal sodium absorption and increased renal sodium retention [37]. In the kidney, proximal tubules can produce and metabolize fructose [42,43], and fructose consumption stimulates renal Na/H activity and sensitization of the renal proximal tubule to angiotensin II—the main effector molecule responsible for blood pressure elevation [44]. This was once an evolutionary advantage to retain water in environments where water was scarce. Yet, in modern times, the overconsumption of fructose due to the high intake of processed foods and sweetened beverages leads to metabolic disorders, including hypertension. In humans, fructose intake causes acute hypertension immediately after consumption and is linked consistently to the rise of chronic hypertension [45,46].

Fructose also leads to increased vasopressin (AVP) secretion, which is one of the main underlying mechanisms of how fructose consumption is thought to lead to metabolic syndrome [47,48]. Given that vasopressin directly promotes renal cyst growth in ADPKD via the vasopressin type 2 receptor (V2R) [49], and given that the sole approved drug for ADPKD is a V2R antagonist [50], it seems clear that fructose consumption cannot possibly be beneficial for individuals with ADPKD but can only be detrimental.

Preclinical studies show that the consumption of a high-fructose diet leads to the development or acceleration of CKD: In rats, fructose-induced hypertension exacerbated renal damage, with high fructose intake causing increased renal sodium retention and sensitivity of the renal proximal tubule to angiotensin II [51,52]. In a rat model of progressive renal failure, a high fructose diet resulted in increased renal size and reduced renal function [53]. In a mouse model of metabolic syndrome and obesity, high fructose corn syrup (HFCS) administration led to rapid CKD development, loss of intrarenal mitochondria, oxidative stress, and increased mortality [54].

Beyond hypertension, excess fructose consumption poses significant health risks comparable to the impacts of alcohol and tobacco [55]. Central to these risks is fructose’s role as a mitochondrial toxin, generating reactive oxygen species, increasing uric acid production (see below), and promoting lipid accumulation, all of which impair mitochondrial function and biogenesis.

Collectively, the above evidence underscores the various deleterious effects of excessive consumption of carbohydrates and sugar/fructose on renal function, providing a high degree of certainty that it is a driver of ADPKD disease progression. No clinical study has ever shown any benefit of a high-carbohydrate diet for ADPKD. Therefore, high-carbohydrate diets—such as the SAD, Mediterranean diet, or DASH diet—should not be recommended to individuals with ADPKD.

### 5.3. Carbohydrate Restriction and Renal Health

Given that chronic kidney “disease” is actually toxicity due to the consequences of persistent hyperglycemia, medical logic should dictate that the first course of action of any healthcare practitioner should be to eliminate any further toxic exposure by placing the patient on dietary carbohydrate restriction. Surprisingly, this simple logic is not reflected in clinical guidelines, neither for diabetic nephropathy nor for ADPKD. Even though the evidence clearly links excess carbohydrate intake to poor kidney health in numerous ways, the dietary recommendations for individuals with ADPKD, still to this day, lack accurate guidance on the intake of carbohydrates. Very high (55%) carbohydrate diets are even recommended as “the PKD diet” based on expert opinion [56] despite the lack of clinical evidence. The burden of proof of benefit is not on ancestrally appropriate dietary patterns but on novel foods and modern dietary patterns such as the very high-carbohydrate SAD.

Part of the problem may be the widespread, yet unfounded, belief that a diet restricted in carbohydrates has deleterious impacts on the human body. Contrary to popular belief, carbohydrates are not essential nutrients for humans, and their consumption is therefore not required. Humans can thrive on a zero-carbohydrate diet because all needed carbohydrates can be synthesized by gluconeogenesis from the glucogenic amino acids contained in dietary protein and from the glycerol backbones of lipids.

Numerous clinical studies have shown that metabolic syndrome, including hypertension, obesity, TD2, and CKD, can be significantly improved and even reversed in patients placed on a more ancestrally appropriate, carbohydrate-restricted diet. This approach is also referred to as ketogenic metabolic therapy (KMT) [57,58,59,60,61,62,63]. We have recently discussed the clinical evidence supporting the efficacy of KMT in CKD, including ADPKD, and refer the reader to these articles for details [62,63].

KMT can utilize a ketogenic diet, which significantly restricts the daily consumption of carbohydrates (20–50 g/day) while protein intake is moderate and fat intake is increased to meet energy needs. This causes a strong reduction in insulin levels, which permits fatty acid release by adipocytes and their conversion to ketones by the liver. The main ketone, beta-hydroxybutyrate (BHB), becomes the main energy source for nearly all cells and tissues during carbohydrate restriction. The entire process of replacing glucose with ketones as energy supply is referred to as “ketosis” and is a normal physiological process (not to be confused with the similar-sounding but unrelated, pathological state of “ketoacidosis”). Ancestral hunter-gatherers, who consumed predominantly low-carbohydrate animal-based foods and did not have around-the-clock access to food, would have been very frequently in ketosis throughout their lives [2,64,65,66]. This is in contrast to today’s humans in industrialized societies, who typically consume frequent meals with high glycemic load throughout the day, which leads to permanent suppression of ketosis, often throughout nearly the entire life of the person. Even short-term suppression of ketosis causes increases in biomarkers of metabolic syndrome, inflammation, and aging [67,68]. Besides ketogenic diets, KMT can also utilize various forms of fasting, such as time-restricted eating and intermittent fasting.

Interestingly, KMT can also be accomplished pharmacologically using the antihyperglycemic SGLT2 inhibitors, which were developed for the treatment of T2D and are now increasingly used in diabetic nephropathy [69]. The mechanism of action of SGLT2i is the inhibition of renal reabsorption of glucose, which leads to daily urinary excretion of up to 80 g of glucose and induces a state of low-level, intermittent ketosis [70]. Mechanistically, the renoprotection of SGLT2i against diabetic kidney disease has clearly been demonstrated to depend on ketone production and their inhibition of mTOR signaling [70]. This supports the idea that a reduction of toxic levels of glucose and induction of ketosis is an effective treatment for kidney disease. However, the same effect could simply be reached by helping the patient to consume 80 g fewer carbohydrates per day instead of using medications that financially burden the healthcare system and have drug-induced adverse effects. A recent study found that SGLT2i treatment led to increased eGFR in subjects with diabetic CKD but only in patients with ketonuria, not in those without ketonuria [71]. This is consistent with the mechanistic studies showing that ketosis is required for the renoprotective effect of SGLT2i.

Similarly, a study on semaglutide in patients with T2D and CKD showed significant benefits, including a 24% reduction in major kidney disease events and a slower decline in eGFR [72]. Semaglutide, originally developed to control T2D but has since gained popularity as a weight-loss drug, acts as a GLP-1 receptor agonist to improve insulin secretion, reduce glucagon levels, slow gastric emptying, and decrease appetite. The renoprotective effects are likely due to improved glycemic control, weight loss, and potential anti-inflammatory mechanisms. However, these benefits could, again, be achievable through reduced carbohydrate intake and KMT, reducing the need for expensive medications with significant potential side effects.

KMT has emerged as an effective treatment for ADPKD. We and others first showed this in several orthologous mouse models of PKD in which time-restricted feeding significantly slowed disease progression [73,74]. We subsequently showed that the beneficial effects depend on the induction of ketosis, which can be induced by several forms of nutritional intervention (time-restricted feeding, fasting, and ketogenic diet) in mouse, rat, and cat models of ADPKD [75]. A ketogenic diet is arguably the most effective treatment of animal models of PKD identified to date. The effect size surpassed that of known pharmacological treatments and was shown to even lead to partial reversion of fully established cystic disease in a rat model [75].

Surprisingly, supplementation with the ketone BHB alone (without carbohydrate restriction or any food restriction) mimicked the beneficial effects of nutritional KMT on ADPKD progression [75,76,77], suggesting that BHB itself underlies the renoprotective effect. This finding is consistent with the above-mentioned mechanism of efficacy of SGLT2 inhibitors in CKD, which also depends on BHB [70], and suggests that direct supplementation with exogenous BHB may be superior to treatment with SGLT2i because the achievable blood BHB levels can be higher and better controlled with BHB supplementation, and because exogenous BHB supplementation lacks adverse drug effects. A medical food for the management of CKD (KetoCitra^®^) containing BHB is already in clinical use [78] and is being tested in ADPKD and other forms of CKD in several ongoing and upcoming clinical studies. The molecular mechanisms underlying the efficacy of BHB in CKD are emerging and are under further investigation. Interestingly, BHB is not only an energy carrier but also a signaling molecule that acts on multiple cellular pathways and has potent effects on cell metabolism, proliferation, and inflammation [79,80,81]. We recently showed that renoprotective mechanisms of BHB include effects on mTOR, GSK-3β, PGC1α, and Nrf2 [77]. It is likely that additional mechanisms are involved, such as known effects of BHB on histone deacetylases (HDACs), the NLRP3 inflammasome, cAMP signaling through the BHB receptor GPR109a, mitochondrial biogenesis and function, and the potent anti-inflammatory effects of BHB [76,77].

In clinical studies, KMT has already shown promise in ADPKD. A retrospective study on 131 individuals with ADPKD who implemented KMT on their own initiative for an average duration of 6 months found feasibility and safety of the intervention as well as beneficial effects on body weight, pain levels, hypertension, and even increased renal function [81]. A ketogenic dietary intervention program for individuals with ADPKD (Ren-Nu.org (accessed on 26 August 2024)) has been available for about two years and combines nutritional intervention with the use of the medical food KetoCitra^®^ to provide exogenous BHB. A report described the program’s feasibility and initial qualitative experience [78]. Larger cohort outcomes will be reported soon, and prospective, controlled studies are underway or upcoming. The first randomized, controlled trial (“KETO-ADPKD”) comparing the ketogenic diet vs. repeated water fasting vs. control was recently reported [82]. In this study, 66 individuals with ADPKD were randomized into 3 groups for 3-month regimens plus a washout period. The ketogenic diet intervention was not only found to be safe and feasible but, surprisingly, resulted in a statistically significant improvement in renal function [82]. A post-hoc subgroup analysis of the KETO-ADPKD trial showed a statistically significant decrease in total kidney volume among subjects in the ketogenic diet arm with the highest BHB levels [82]. A multi-center prospective observational cohort study of 521 participants with ADPKD who were not undergoing KMT found that higher BHB blood levels were associated with slower kidney function decline [83]. This suggests that BHB, even at levels below those found in ketosis, may have renoprotective effects and further emphasizes the potential utility of raising blood BHB levels through nutritional means of BHB supplementation.

As a practical matter, replacing sugar with non-nutritive sweeteners may appear like an attractive way of reducing sugar intake and supporting dietary interventions aimed at KMT. We caution, however, that many artificial sweeteners may have potential adverse effects on kidney health. One possible exception may be natural sweeteners based on stevia, which were reported to not only be benign to kidneys at very high administered doses but potentially have beneficial effects in human CKD studies [84] and rodent models of ADPKD [85,86].

Collectively, the above evidence shows that carbohydrate overconsumption and glucose toxicity act as triggers that worsen ADPKD progression. Conversely, carbohydrate restriction, KMT, and BHB supplementation show excellent promise as disease-modifying measures to slow, stop, or potentially even partially reverse ADPKD progression. Clearly, individuals with ADPKD should not be advised to remain on diets with very high glycemic loads that have no evidence of benefit but likely do harm based on current knowledge.

## 6. Tubular Microcrystals as Triggers of Cystogenesis

As a major detoxifying organ, the kidneys are constantly challenged with waste products and toxins that are poorly soluble and tend to precipitate to form micro-crystals in tubule lumens during the concentration of the primary filtrate. In humans, the most common natural examples are calcium oxalate, calcium phosphate, and uric acid. If not cleared, these micro-crystals can go on to form kidney stones. The same applies to artificial toxins, including many medications, that can precipitate, leading to nephrotoxicity (see further below).

**Figure 3:**
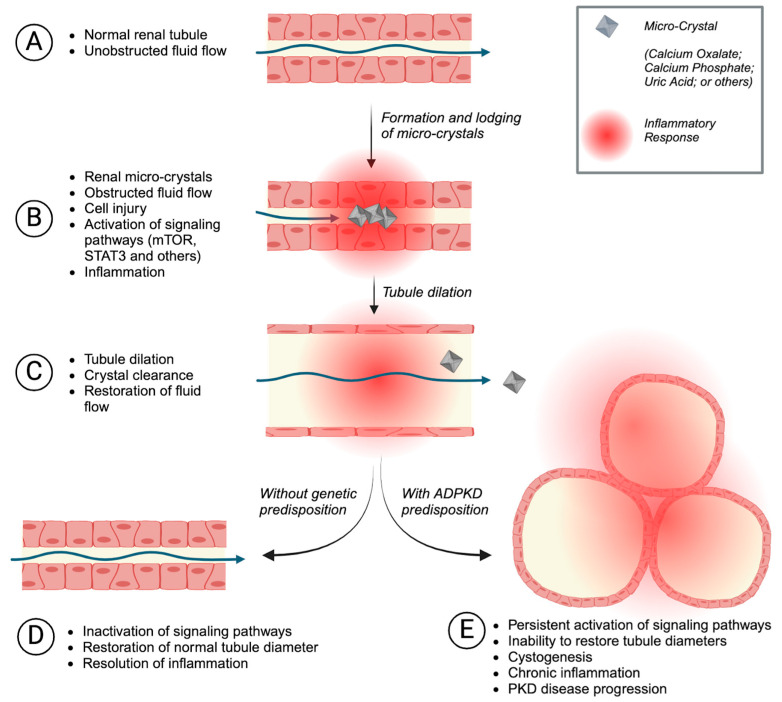
**Renal crystal-mediated injury, inflammation, tubule dilation, and ADPKD progression**. A normal renal tubule with unobstructed fluid flow (**A**). Diet, dehydration, or medication-induced deposition of microcrystals (most commonly consisting of oxalate, uric acid, or phosphate) causes obstruction of renal fluid flow, renal injury, and inflammation (**B**). The renal tubule dilates to allow for crystal clearance and to re-establish normal fluid flow (**C**). Without any genetic predisposition, temporary microcrystal injury, inflammation, and tubule dilation are resolved (**D**). In ADPKD patients, crystal-mediated inflammation and injury can initiate or accelerate renal cyst growth and disease progression (**E**).

We have previously shown that renal tubular micro-crystal formation triggers cystogenesis in genetically preconditioned PKD rodent models [22]. The underlying mechanism appears to be caused by a previously unrecognized renoprotective response to tubular micro-crystals (Figure 3). We demonstrated that lodged micro-crystals cause activation of certain signaling pathways, leading to tubule diameter dilation, which then allows the affected tubule to flush out the lodged crystals (Figure 3A–C) [22]. In healthy kidneys, this process is reversible once crystals are successfully cleared (Figure 3D). In genetically susceptible kidneys, however, tubule dilation continues and eventually leads to cyst formation (Figure 3E), thereby worsening the progression of PKD [22]. Chronic exposure to renal micro-crystals can lead to renal cystogenesis even without PKD mutations, as has recently been shown in genetic disorders characterized by chronic nephrocalcinosis [87,88]. Altogether, these results suggest that kidneys generally respond to tubular micro-crystals by activating a tubule dilation response, and that individuals with ADPKD are particularly vulnerable because this response triggers cyst formation in their predisposed kidneys.

Individuals with ADPKD are more prone to micro-crystal formation and kidney stones because they frequently have an abnormally low urine pH due to metabolic acidosis [89,90]. Low urine pH increases the risk of uric acid precipitation [91]. Low urine pH also causes hypocitraturia, and individuals with ADPKD consequently have abnormally low urine citrate levels [22,92], which increases the risk of calcium crystal precipitation. Therefore, kidney stone disease is more common in individuals with ADPKD than in the general population [93].

The prevalence of kidney stones has dramatically increased worldwide during the past few decades and is strongly associated with poor metabolic health and obesity [94,95]. Calcium oxalate kidney stones are by far the most common type of stones, followed by uric acid and calcium phosphate stones. All are primarily induced by dietary factors, and we will discuss them separately below.

### 6.1. Oxalate

Oxalate is a natural metabolic waste product (e.g., from the catabolism of glyoxylate), is endogenously produced at a somewhat constant rate, and is primarily cleared via the kidneys [96]. However, the amount of oxalate that kidneys need to clear can peak significantly in response to the ingestion of oxalate-rich foods, which can lead to a form of acute kidney injury that can transition into CKD, termed oxalate nephropathy [97,98,99]. Most plants contain oxalate, which they use as an anti-nutrient and defense mechanism. This renders most plants poisonous to humans. Even many “edible” plants are indeed quite toxic to humans due to their high oxalate content. Well-known examples are spinach, Swiss chard, and many other leafy greens, rhubarb, almonds, and many other nuts, dragon fruit, star fruit, soy, beets, cocoa, potatoes, grains, and countless other plants and their processed products. The literature is full of case reports of oxalate nephropathy caused by ingestion of foods such as nuts and seeds [100], star fruit [101], and popular novel foods that are hailed as healthy (without evidence), such as fruit/vegetable juices or green smoothies [102,103]. If excessive ingestion of oxalate-rich foods can cause full-blown acute kidney failure, it stands to reason that even the ingestion of lesser amounts may cause subclinical tubular injury that would ultimately be harmless to individuals with healthy kidneys but may be detrimental to individuals with ADPKD and result in worsened disease progression.

Citrate is the normal defense of kidneys against calcium crystal precipitation because of the ability of citrate to chelate calcium and prevent its binding to oxalate [104]. Hypocitraturia is common in individuals with ADPKD due to the abnormally low urine pH. We and others reported that hypocitraturia is strongly associated with faster ADPKD progression, suggesting a causal link via the mechanism of tubular injury caused by calcium micro-crystals illustrated in Figure 3 [22,92]. Indeed, supplementation with citrate potently inhibits not only renal calcium crystal accumulation but also cystic disease progression in a rat model of PKD [22,76].

We suggest that individuals with ADPKD should be advised to reduce their calcium oxalate lithogenic risk by (1) reducing their intake of oxalate-rich foods, (2) consuming enough calcium with meals to bind dietary oxalate, (3) normalizing (raising) their urine pH level, and (4) normalizing their urine citrate level. Since this may be difficult to accomplish by dietary changes alone, the incorporation of supplements or medical foods aimed to address these issues may be required.

### 6.2. Phosphate

While phosphate is crucial for numerous physiological processes, maintaining balanced phosphate levels is critical, as excessive intake can lead to the deposition of calcium-phosphate crystals within renal tubules, causing acute kidney injury and phosphate nephropathy [105,106]. The use of oral sodium phosphate solutions (OSP), commonly prescribed as laxatives before medical procedures, exemplifies this risk, particularly in individuals with pre-existing kidney conditions or those at higher risk for renal dysfunction [105]. OSP can significantly increase blood phosphate levels, exceeding what the kidneys can efficiently process and leading to phosphate nephropathy, which is well documented in the medical literature [107].

Overconsumption of phosphate-rich foods and beverages may also pose a significant health risk. In Western diets, a major source of dietary phosphate load originates from inorganic phosphate food additives used for preservation and flavor enhancement [108], commonly found in highly processed foods such as fast foods, canned soups, and frozen dinners. The phosphorus bioavailability of these food additives is higher than that of whole foods [109]. Thus, excessive phosphate intake is common in industrialized countries and often surpasses physiologically tolerable levels in many individuals. This is particularly a concern in the context of individuals with ADPKD who may experience altered phosphate handling due to decreased renal function. Similar to the above-mentioned effects of oxalate exposure, we reported that merely doubling the dietary phosphorus intake induced renal tubular calcium phosphate deposition, leading to strongly accelerated disease progression in a PKD rat model [22]. A subsequent clinical study found a significant association between serum phosphate levels and poor renal prognosis in patients with ADPKD [110]. A similar association is already well documented for CKD in general and was similarly linked to calcium phosphate crystal-mediated tubular injury [106]. An elevated level of serum FGF23, the hormone triggering increased urinary phosphate excretion, appears to be a sensitive predictor of progression in CKD [106].

Given these data, given that most individuals in industrialized societies over-consume phosphorus due to inorganic additives in processed foods, and given that inorganic additives have no nutritional benefit, the responsible advice to any patient with ADPKD should be to manage their phosphate intake carefully by reducing the consumption of processed foods and beverages containing phosphate additives. Additional steps are ensuring a sufficient intake of magnesium and calcium that can help mitigate phosphate absorption and monitoring and managing blood phosphate levels.

### 6.3. Uric Acid

Uric acid is the end product of the metabolic breakdown of purines, which are ubiquitous in the body and in many foods, as components of nucleic acids (DNA and RNA) and nucleotides (e.g., ATP) [111]. Normally, uric acid is produced at a relatively constant rate due to normal purine catabolism, predominantly processed by the liver and excreted by the kidneys [111]. However, uric acid levels can significantly increase from the catabolism of either fructose or ethanol by the liver. Fructose, consumed in the form of added sugar (sucrose) or high-fructose corn syrup, or as natural sugar in fruits and juices, is metabolized primarily by the liver, leading to ATP consumption and its degradation to uric acid [112]. The same is true for alcohol [113]. Ancestral humans consumed an insignificant amount of sugar/fructose, maybe a few grams per day, in stark contrast to modern sugar consumption, which can exceed 100 g per day due to the prevalence of added sugars in processed foods and beverages.

Hyperuricemia, the accumulation of high uric acid levels in the blood, can lead to gout, characterized by painful joint inflammation due to sodium urate crystal deposits [114]. Hyperuricemia can also promote the precipitation of uric acid micro-crystals in renal tubule lumens and lead to the formation of uric acid kidney stones [115]. Renal tubular uric acid precipitation is not only driven by elevated serum uric acid concentration but also by low urine pH due to the decreased solubility of uric acid in acidic environments.

The relationship between high uric acid levels and kidney health is particularly concerning in individuals with ADPKD. In ADPKD, compromised kidney function can exacerbate the difficulties in uric acid clearance, leading to higher risks of uric acid kidney stone formation and accelerated progression of kidney disease [116]. Several studies have shown that high levels of serum and urinary uric acid are indicators of poor outcomes in ADPKD [117,118,119]. For instance, a study found that higher serum uric acid levels were significantly associated with earlier onset of hypertension, increased total kidney volume (TKV), and a higher risk of progression to ESRD in ADPKD patients, independent of other factors such as gender, BMI, and renal function [117]. In contrast, a retrospective study based on HALT PKD data concluded that serum uric acid levels were not an independent predictor of disease progression when adjusted for baseline eGFR [120]. However, this study showed that serum uric acid correlates with an increase in TKV in men. Differences in methodology may explain the discrepancy. For example, the latter study only had serum uric acid data available from a single time point during the multi-year trial and did not investigate urinary excretion of uric acid [120]. A recent study showed that while serum uric acid is a poor predictor of chronic kidney disease (CKD) progression, urine uric acid concentration strongly correlates to disease progression [121]. This finding offers a potential resolution to the controversy by highlighting the importance of urine uric acid levels in understanding and predicting CKD and ADPKD progression. It is also consistent with a mechanism in which renal tubular uric acid microcrystals worsen ADPKD progression due to crystal-induced cell injury.

Given the potential risks associated with a high burden of renal uric acid excretion, it is reasonable to routinely monitor serum uric acid and urine pH levels in individuals with ADPKD. High serum uric acid levels in combination with acidic urine pH represent an increased risk for the precipitation of damaging uric acid micro-crystals during renal excretion. Preventative measures to reduce this risk with dietary and lifestyle changes include: (1) reducing intake of high-fructose foods and beverages, (2) reducing alcohol consumption, (3) patient self-monitoring of urine pH and preventing excessive and prolonged urine acidification, (4) reducing excessive consumption of high-purine foods, and (5) maintaining adequate hydration to facilitate uric acid excretion. These management strategies aim to reduce the risk of renal injury from uric acid micro-crystals and the formation of kidney stones, which may, therefore, slow the progression of ADPKD and also minimize the risk of gout.

### 6.4. Dehydration

Dehydration greatly increases the risk for any type of kidney stone and, therefore, the risk of forming renal tubular micro-crystals of all kinds. Given that micro-crystals worsen ADPKD disease progression, as discussed above, it follows that dehydration can only be detrimental in ADPKD. Treatment of individuals with ADPKD with tolvaptan greatly induces thirst, fluid intake, and polyuria, thereby diluting solutes during passage through the kidneys and decreasing the lithogenic risk [122]. Besides inhibiting vasopressin signaling, it seems likely that the suppression of tubular micro-crystal precipitation by urine dilution may be a major mechanism underlying the efficacy of tolvaptan.

Increased water intake significantly slows disease progression in rat models of ADPKD [123,124]. Consistent with this, a small cross-over clinical trial in patients at risk of rapid progression of ADPKD found that high water intake slowed the rate of TKV growth and reduced pain [125]. A larger, 3-year randomized trial, however, found no benefit of increased water intake [126]. Differences in study design and execution likely explain the disparate outcomes. For example, the control group in the larger trial [126] also increased their fluid intake to nearly the same level as the intervention group, which likely prevented the detection of any possible benefit. This illustrates the difficulties of using randomized control groups in trials involving diets or—in this case—water because blinding is impossible, and it becomes very difficult to prevent subjects in the control group from implementing the intervention on their own accord. Despite the unsatisfactory consensus of clinical studies on water intake, the results from animal studies together with biological mechanistic plausibility and common sense lead to the conclusion that sufficient hydration is an important recommendation for individuals with ADPKD. Certainly, patients should be urged to avoid dehydration with high priority.

### 6.5. Medications and Dietary Supplements

Adverse drug reactions (ADRs) are a leading cause of death worldwide [127,128] and have been argued to be the number one leading cause in the US. Nephrotoxicity is one of the common ADRs. Specific nephrotoxic effects of medications are discussed in other sections of this review. In this section, we focus specifically on the connection between certain medications or supplements and the development of kidney crystals and stones. Renal tubular micro-crystals caused by ADRs should be considered as equally detrimental to the progression of ADPKD as micro-crystals resulting from dietary factors discussed above.

Medications can cause renal injury by crystallizing directly in renal tubule lumens or by inducing changes in urine chemistry that favor crystal and stone formation [129]. For example, antibiotics like sulfonamides or vancomycin can lead to the formation of renal tubular sulfonamide [130] or vancomycin [131] precipitates, respectively, causing AKI. Diuretics, particularly loop diuretics and thiazides, can alter urine composition by increasing calcium excretion or decreasing citrate excretion, both of which can contribute to the formation of calcium oxalate or phosphate stones [129]. Medications used for treating bacterial infections and HIV, such as protease inhibitors, can also induce changes in urine chemistry, leading to the formation of both radiopaque and radiolucent stones [132]. Additionally, osmotic laxatives containing PEG 3350 pose risks due to potential contamination with ethylene glycol and diethylene glycol, substances that are metabolized to oxalate and increase the risk of forming calcium oxalate crystals [133]. These examples underscore that the use of any medication should be particularly carefully considered for individuals with ADPKD, especially if the medication is prescribed long-term.

Dietary supplements, too, can contribute substantially to renal crystal and stone formation. Per FDA regulations, dietary supplements are not intended to treat or prevent any diseases but are only intended for the maintenance of health in healthy individuals. Therefore, by definition, there are no supplements “for” kidney disease. Furthermore, dietary supplements do not have to be GRAS (generally recognized as safe) because they are not regulated as foods or medical foods. Consequently, dietary supplements can contain almost any substance or even completely undefined mixtures of thousands of substances, such as plant extracts.

Most consumers and healthcare practitioners often lack a clear understanding of the potential dangers of ingredients in dietary supplements, especially for individuals with ADPKD with potentially compromised renal function that may greatly exacerbate these dangers. High-dose vitamin C, for example, can increase the risk of oxalate nephropathy and stone formation due to its breakdown into oxalate [98]. Similarly, high-dose vitamin D supplementation may worsen the risk for stone formation in patients predisposed to hypercalciuria [134]. Additionally, many herbal and plant-based supplements—such as “green superfood” supplements, beetroot powders, turmeric and curcumin supplements, green tea extracts, and many others—can contain high amounts of oxalate and may potentially endanger individuals with ADPKD. A reasonable general recommendation for individuals with ADPKD would be to avoid any dietary supplements unless necessary to prevent an established or suspected micronutrient deficiency that cannot be addressed by dietary changes, and unless the ingredients in any given supplement are carefully scrutinized by an experienced expert.

## 7. Sodium and Potassium

Sodium and potassium are essential for most physiological processes and must be obtained through diet. Changes to our modern food system towards more processed and storable food items have dramatically increased daily consumption of sodium salts while simultaneously reducing the intake of potassium salts. Shelf-stable foods require transportation to reach markets, increasing the need for added preservatives, most of which are sodium salts (e.g., chloride, benzoate, sorbate). Ancestral diets were estimated to contain a 1:10 molar ratio of sodium/potassium, whereas modern diets are estimated to contain a 3:1 molar ratio of sodium/potassium [135].

In preclinical PKD models, it has been well demonstrated that increased sodium intake can significantly accelerate cystic disease progression [76,136]. Similarly, potassium restriction has been shown to accelerate PKD progression in animal models [137]. This relationship between sodium vs. potassium intake has also been observed in human clinical studies of ADPKD. Urine sodium excretion was found to positively associate, and urine potassium excretion to negatively associate with disease progression [138,139]. This observation was extended and confirmed by Kramers et al., who found again that sodium intake was associated with worse disease outcomes [140].

While the risk of excessive potassium in late-stage CKD has been well documented, many studies highlight the importance of potassium before late-stage kidney disease for preserving and maintaining healthy kidney function. A meta-analysis of chronic kidney disease (CKD) prognosis found that both high and low serum potassium were predictors of adverse outcomes [141]. Low potassium levels are associated with increased blood pressure and sodium retention, whereas potassium supplementation using chloride or citrate salts lowers blood pressure in healthy individuals [142]. Urinary potassium excretion, an estimate for dietary intake, is associated with a lower risk for CKD in healthy individuals [143]. Similarly, higher urinary potassium in diabetic patients is associated with lower odds of renal replacement [144].

Altogether, the sodium/potassium ratio may be most important as opposed to the absolute intake values of each electrolyte. Sodium promotes fluid retention, increasing blood volume and blood pressure, thereby exacerbating renal stress and may worsen chronic kidney disease (CKD). Conversely, potassium has a protective role in both hypertension and renal health. Higher potassium intake helps mitigate the adverse effects of sodium, enhances sodium excretion, and promotes vasodilation, which collectively contributes to lower blood pressure and reduced renal stress [145].

These considerations suggest that the inverted ratio of sodium vs. potassium in modern processed diets, as opposed to an ancestrally appropriate ratio, contributes to the worsening of ADPKD disease progression.

## 8. Increased Intestinal Permeability

Increased intestinal permeability, commonly referred to as “leaky gut”, involves the partial loss of intestinal barrier function, allowing the passage of substances—including entire bacterial cells—from the inside of the gastrointestinal tract through the epithelial layer lining the intestinal wall into the circulation and rest of the body [146,147]. Substances that can potentially cross the disrupted intestinal barrier may be derived from ingested food that is metabolized by the intestinal microbiome or may be toxins, such as the bacterial cell wall component lipopolysaccharide (LPS), that emanate from intestinal bacteria directly. These substances may increase chronic inflammation throughout the body. Since the kidneys constantly filter a large volume of blood, it is reasonable to assume that kidneys are particularly vulnerable to the effects of increased intestinal permeability. Modern diets, especially components in ultra-processed foods, can disrupt the intestinal barrier in several ways [148,149]. These include (1) direct chemical irritation of the intestinal wall by common food additives (emulsifiers, preservatives, artificial sweeteners, HFCS), (2) alterations in the gut microbiome, and (3) immune responses to components of modern foods such as that to wheat gluten (Celiac disease) and certain plant fibers (inflammatory bowel diseases) [150,151].

The relationship between ADPKD, the microbiome, and microbial products was initially shown decades ago [152]. A PKD mouse model kept in a sterile environment did not develop cystic disease compared with mice kept in an ambient environment, suggesting a role of intestinal bacteria in cystogenesis [153]. Another study showed that oral administration of the nephrotoxin nordihydroguaiaretic acid (NDGA) alongside endotoxin-containing bacteria could initiate cyst formation synergistically in otherwise germ-free mice [154]. Similarly, NDGA-induced renal disease was accelerated significantly after exposure of germ-free rats to a non-sterile environment [154]. More recently, the presence of uremia in both rats and patients with ESRD was associated with profound alterations in gut microbiota, and, in ADPKD patients specifically, the gut microbial composition changed according to renal function [155,156,157]. Analysis of ADPKD cyst fluid found the presence of both endotoxin and fungal products, implicating microbial-derived products as potential triggers for cyst formation [152].

Many of the dietary and environmental triggers discussed in this article are known gut and gut microbiome disruptors, including fructose, common food additives in ultra-processed foods, many medications (e.g., antibiotics), smoking, and alcohol [158,159,160]. Individually and collectively, these are likely triggers that may worsen the progression of ADPKD. Resolving intestinal barrier disruptions should be an important part of the management of individuals with ADPKD. A potential strategy may be the use of probiotics. Probiotics are gaining attention as a potential adjunctive therapy in CKD due to their possible ability to improve gut health, reduce systemic inflammation, modulate the gut microbiome, and lower uremic toxin levels [161,162,163,164]. While probiotics show promise in promoting gut–kidney axis health, more robust clinical trials are needed to better understand their potential efficacy in CKD. To our knowledge, no human studies on the effects of probiotics in ADPKD are currently available.

## 9. Nephrotoxins

The kidneys not only eliminate metabolic waste products but are also instrumental in the detoxification of artificial and natural toxins. However, this critical role also exposes the kidneys to potential harm and makes them vulnerable to the adverse effects of these substances. Exposure to such toxins can lead to nephrotoxicity, which may lead to AKI and, eventually, CKD [165]. Numerous nephrotoxins are well-known inducers of CKD, and several have been shown to worsen the progression of ADPKD. We consider it likely that any significant exposure to a nephrotoxin has a high potential to trigger cystogenesis and hasten disease progression in individuals with ADPKD. Important classes of nephrotoxins that patients in industrialized societies are exposed to regularly are discussed below.

### 9.1. Medications

In previous sections, we discussed the specific ways certain medications and dietary supplements contribute to renal crystal and stone formation. We highlighted how the crystallization of these substances in urine or their impact on urine chemistry can lead to significant renal complications, particularly in individuals with a predisposition to kidney stones or conditions such as ADPKD. However, the nephrotoxic effects of medications can extend beyond their role in crystal formation. In this section, we broaden our discussion to encompass the general impact of medications on renal health and focus on specific examples.

#### 9.1.1. NSAIDs

NSAIDs are a class of medications widely used to reduce inflammation, alleviate pain, and lower fevers. Commonly known examples include ibuprofen, naproxen, and aspirin. They work by inhibiting enzymes involved in the inflammatory process and effectively manage symptoms associated with conditions like arthritis, menstrual pain, and headaches [166]. Yet, particularly in susceptible individuals, NSAIDs can have significant side effects, affecting the gastrointestinal tract and kidneys through mechanisms including the reduction of blood flow, decreased prostaglandin synthesis, and direct toxicity [166]. Given that pain is a common complication in individuals with ADPKD, there is a frequent desire for analgesics among this population [167,168,169,170]. NSAIDs are frequently available without prescription, do not have prominent toxicity warnings, and are commonly considered benign among lay people. This increases the chance of inadvertent worsening of disease progression among individuals with ADPKD.

The impact of NSAIDs on renal health has been studied extensively, and chronic use of NSAIDs is well-established as a risk factor for the development of CKD. Particularly, ketorolac and oxicams, like meloxicam and piroxicam, may increase the risk of CKD due to their potential to cause acute renal damage when used over a long period [171]. Due to this and many such examples, the use of NSAIDs in individuals with ADPKD is generally discouraged. Interestingly and somewhat contradictory to these associations, selected preclinical studies recently proposed that NSAIDs, due to their inhibition of prostaglandin synthesis, may potentially slow cyst development. Treatment with Sulindac reduced renal cyst burden in a mouse model of PKD, and treatment with acetylsalicylic acid had similar effects in a rat model of PKD [172,173]. While this is an interesting emerging avenue for potential future therapeutic targets, it remains to be clinically shown if (1) the selected inhibition of prostaglandins is also effective in human ADPKD and if (2) the potential beneficial effects outweigh the known nephrotoxic effects of long-term NSAID use [174]. Thus, as the evidence stands to date, caution and regular renal monitoring are advisable when considering NSAID use in ADPKD patients, with a preference for alternative pain management strategies whenever possible.

#### 9.1.2. Antibiotics

Antibiotics are widely used to treat bacterial infections. They are among the most commonly prescribed medications globally and are crucial tools in medicine for treating infections and preventing the spread of disease. Infections of the urinary tract, including the kidneys and renal cysts, are known complications in ADPKD patients, potentially impacting their overall renal health and disease progression. The presence of cysts can obstruct urinary flow and create environments conducive to bacterial growth. Antibiotics are effective treatments and are required to prevent recurrent infections. However, adverse effects of frequent antibiotic use include the development of antibiotic resistance, destruction of the gastrointestinal microbiota (dysbiosis, discussed above), and acute kidney injury.

Similar to studies on NSAIDs, the negative impact of chronic antibiotic use on renal health is well established. This is particularly the case for older individuals who, despite lower renal function and reduced efficiency in clearing drugs, are frequently prescribed antibiotics. Consequently, these drugs can accumulate to harmful levels, leading to kidney damage. This scenario was notably observed in elderly patients prescribed aminoglycosides, necessitating reduced treatment durations to mitigate adverse effects [175]. Additionally, as people age, the prescription of medications like antihypertensives becomes more common, and the concurrent use of certain antibiotics, such as trimethoprim-sulfamethoxazole, can result in harmful drug interactions that further compromise renal function [176]. This aspect is crucial for ADPKD patients, who face heightened infection risks and frequent interventions for hypertension, potentially exacerbating side effects that harm kidney function and accelerate disease progression.

Clinical studies that evaluate these nuances of certain drug interactions and, generally, the safety of antibiotics in ADPKD are still lacking. Since antibiotics have a significant effect on kidney function, particularly in individuals already suffering from kidney issues, careful antibiotic selection and dosage are crucial to prevent further renal damage.

### 9.2. Psychoactive Substances

Psychoactive substances affect the brain, influencing mood, consciousness, cognition, and behavior. They can be both naturally occurring and synthetically manufactured. These substances encompass a broad range of drugs, including alcohol, caffeine, nicotine, prescription medications like antidepressants and anxiolytics, and illegal drugs such as cannabis, cocaine, and heroin. In the following, we specifically focus on alcohol and nicotine/cigarette smoking, which are among the most common legal psychoactive substances that have also been linked to CKD and ADPKD progression.

#### 9.2.1. Alcohol

Alcohol is used socially and ceremonially in many parts of the world. It affects the central nervous system as a depressant, altering mood, consciousness, and behavior. Adults in most countries can legally consume it, and its role in social and cultural practices contributes to its widespread use. However, even low levels of alcohol consumption can be linked to many negative health outcomes, which has led to the World Health Organization (WHO) recommendation, “No level of alcohol consumption is safe for our health” [177]. Alcohol affects the kidneys, too, and has been linked to the development of renal disease, particularly due to alcohol-induced hypertension [178]. Furthermore, as discussed in a previous section, alcohol is catabolized by the liver, leading to increased uric acid production, which increases the risk of forming damaging uric acid micro-crystals in renal tubules. The consumption of alcohol is unadvisable for individuals with ADPKD, even though specific studies directly linking alcohol consumption with ADPKD progression are lacking.

#### 9.2.2. Nicotine/Smoking

Nicotine is a stimulant and the primary addictive ingredient in cigarettes, cigars, and other tobacco products, including smokeless tobacco and some e-cigarettes. Nicotine itself stimulates the central nervous system, enhancing mood, cognition, and alertness, but it is also a vasoconstrictor, lowering blood flow in the microvasculature (e.g., kidney nephrons) while raising heart rate and blood pressure. Apart from the effects of nicotine itself, smoking as a form of nicotine delivery poses significant health risks, including an increased likelihood of developing heart disease, lung cancer, and numerous other serious health conditions [179]. Smoking is also widely recognized as a significant risk factor for renal disease, including ADPKD [180]. In CKD patients, smoking is independently associated with disease development along with cardiovascular morbidity and mortality [179]. In ADPKD animal models, as well as clinical studies, smoking was found to accelerate disease progression. In a mouse model of ADPKD, mice exposed to smoke showed elevated cyst lining cell proliferation and increased renal cystic index [181]. The harmful effects of smoking on kidney function are attributed to its impact on renal hemodynamics and its association with other deleterious health effects that compound the risk of kidney disease progression. These include smoking-induced inflammation and oxidative stress, activation of the sympathetic nervous system, glomerular capillary hypertension, endothelial cell dysfunction, and heavy metal toxicity [182]. Thus, existing evidence overwhelmingly identifies smoking as a trigger, and cessation is likely to slow ADPKD progression while also improving overall health. The recommendation to avoid or cease smoking is well accepted as part of the clinical management of ADPKD patients.

### 9.3. Environmental Nephrotoxins

Environmental nephrotoxins are substances found in the environment that cause renal injury. Various sources include industrial chemicals, agricultural pesticides, heavy metals, and organic solvents. Depending on the property of the toxin, ingestion, inhalation, or skin contact may lead to acute or chronic kidney damage. Specific examples of prevalent and naturally occurring nephrotoxins include arsenic, cadmium, lead, and mercury. They are associated with the development of CKD due to their ability to cause oxidative stress and tubular cell apoptosis. Chronic exposure to these metals can lead to a decline in kidney function by impairing the kidneys’ ability to filter blood and excrete waste effectively [165]. Specific examples of manufactured nephrotoxins include common plasticizers, bisphenol A (BPA), and phthalates. These also induce oxidative stress within the kidneys, which can contribute to the progression of CKD. This is particularly the case for sensitive populations like children, who may have more significant exposure due to their behavior and higher food consumption relative to their body weight [183].

Metals and plasticizers are some of the more known examples of nephrotoxins, but the discovery of new sources of nephrotoxins, sometimes in the form of commonly used products, is, unfortunately, a reality. A recent example of this is the discovery that continued exposure to glyoxylic acid, commonly found in products used for hair straightening procedures, leads to oxalate nephropathy in both animals and humans [184]. In some instances, the occurrence of high rates of CKD precedes the discovery of the underlying nephrotoxin(s). This is likely the case for CKD of unknown etiology that primarily occurs in young men in tropical and agricultural countries and which is suspected, but not proven, to be caused by a combination of nephrotoxins and other factors, including heavy metals, agrochemicals, as well as heat, dehydration, hyperuricemia, and silica nanoparticles [185,186,187].

Some of the environmental nephrotoxins described in this review are relatively common, while others occur only in very specific circumstances. Yet, a general understanding of possible risks and attention to newly emerging information may prevent exposures to environmental ‘triggers’, which is especially critical in cases of a predisposition such as ADPKD.

## 10. Probably Not a Trigger: Protein

The idea that dietary protein consumption, beyond an essential amount, may be harmful to human kidneys has been ingrained in the consciousness of the renal health profession despite the lack of compelling evidence. The trend towards recommending protein restriction probably originated from the publication of an influential review article by Brenner et al. that summarized experimental results in various animal species (rats, dogs, desert quails, vampire bats, etc.) [66]. Interestingly, Brenner et al. actually made the point that only ad-libitum consumption of food (e.g., the consumption of many small meals/snacks throughout the day) leads to persistent hyperfiltration, which may harm kidneys. In contrast, Brenner et al. proposed that our hunter-gatherer human ancestors would have only consumed a single large meal every two days, resulting in harmless intermittent hyperfiltration [66]. Despite the fact that Brenner et al. only made a compelling case for intermittent fasting (which would also limit carbohydrate consumption and induce ketosis; see above), their paper was interpreted by generations of renal health professionals as “evidence” that protein intake should be limited to preserve renal function.

There is a common misconception that may have contributed to the belief that protein intake should be restricted for the benefit of renal health. Many authors, including Brenner et al. [66], believe that “Western” diets are “high” in protein content. In fact, the opposite is the case. Protein in Western diets typically accounts for approximately 15% of total calorie intake [188]. In contrast, protein in ancestral diets of pre-agricultural hunter-gatherers is estimated to have accounted for 19% to 35% of total calorie intake [2,5,65,189]. Therefore, if anything, “Western” diets are actually very low in protein content relative to a human species-appropriate diet.

Numerous clinical studies have investigated the possible relationship between protein consumption and renal health, and the results have been meta-analyzed and reviewed extensively without finding any compelling adverse link [190,191,192]. If anything, meta-analyses indicate either no association between CKD risk and protein consumption or a protective effect of higher protein consumption [192]. Even Brenner’s proposed link between hyperfiltration and the risk of developing CKD could not be confirmed in clinical studies [193,194]. A recent large and long-term study found that higher protein intake from plant and animal sources is linked to lower mortality in older adults with CKD [195]. Unfortunately, the recommendation of protein restriction is being perpetuated in renal guidelines to this day [190].

Similar to the lack of an association between protein intake and the risk of general CKD progression, clinical studies have also found no association for ADPKD [140,196]^.^ Numerous studies have been published (which we will not list here) in which rodent models of PKD have been subjected to highly species-inappropriate diets containing extreme amounts of certain proteins or amino acids. These studies reported negative effects on PKD progression. However, the predictive value of these studies for human ADPKD is questionable. Perhaps the only conclusion that should be drawn is that consuming a species-inappropriate diet is not a good idea, neither for mice nor humans. Given that human clinical studies trump animal studies and given that those clinical studies found no association between protein consumption and ADPKD progression, it is reasonable to conclude that protein consumption in a normal range reflecting the ancestrally appropriate level does not act as a trigger of increased progression of ADPKD.

## 11. Conclusions

The recognition that dietary and environmental factors may trigger the progression of genetically inherited disorders like ADPKD represents a promising and evolving area of research. Until now, most individuals diagnosed with ADPKD are told that their disease is relentlessly progressive and that there is nothing they can do to change this fate. Considering everything we know today, this still common fatalistic medical advice is outdated and counterproductive. The insights discussed in this review suggest a paradigm shift: chronic kidney disease in ADPKD may not only be manageable but potentially preventable through disease-modifying, conservative, and very accessible dietary and lifestyle modifications, which also enhance overall health. Many individuals with ADPKD are highly motivated to try their best to change the course of their disease, especially with diet and lifestyle modifications that are under their own control. This provides an opportunity for healthcare practitioners to induce their patients to make beneficial choices that have a chance to lead to tangible improvements in disease outcomes and their general health and well-being. None of these diet and lifestyle choices require approval by the FDA or any guidelines. Individuals with ADPKD have autonomy and are free to consume the foods they want, and to avoid whichever toxins they want to avoid. However, patients require—and expect—expert advice from their well-informed healthcare practitioners. Any healthcare practitioner is free to recommend conservative diet and lifestyle choices to their patients. Medical practitioners are not required to restrict their professional armamentarium solely to the prescription of medications.

This is an evolving topic, and the list of potential triggers outlined here is undoubtedly incomplete and subject to future revisions. For instance, while dietary protein was once thought to exacerbate ADPKD, newer studies have changed this view. Conversely, novel triggers, such as emerging new forms of pollution, which were not discussed due to the current lack of sufficient evidence, may later prove significant in ADPKD progression. This underscores the necessity for nephrologists and patients to stay informed about emerging research and to adapt dietary and lifestyle recommendations accordingly. While such adjustments might initially seem daunting, adhering to simple guidelines can significantly benefit patients. Reasonable advice to individuals with ADPKD should at least include these main points: (1) choosing whole, unprocessed foods, (2) maintaining proper hydration, (3) avoiding known harmful substances, including food additives, toxins, supplements and medications, (4) understanding food composition (e.g., oxalate or phosphate levels) and its impact on kidney health, (5) substantially reducing carbohydrate (sugars and starches) intake and incorporating forms of ketogenic metabolic therapy such as time-restricted eating, intermittent fasting, ketogenic diets or BHB supplementation, and (6) promoting intestinal health.

Armed with these principles, individuals with ADPKD can potentially delay or avoid life-impacting treatments, dialysis, or transplantation, thereby markedly improving their health and quality of life. This comprehensive perspective of ADPKD management emphasizes the urgent need for updated clinical guidelines that integrate conservative diet and lifestyle changes, offering a hopeful outlook for patient outcomes.
